# Preparation and Characterization of Polydatin–Chitosan Nanocapsules for Enhanced Drug Delivery Efficacy

**DOI:** 10.3390/molecules30224400

**Published:** 2025-11-14

**Authors:** Donato Nichil, Sofia Migani, Marisa Colone, Leonardo Severini, Simona Sennato, Giuseppina Bozzuto, Aurora Patrizi, Cecilia Bombelli, Giampietro Ravagnan, Annarita Stringaro, Leonardo Mattiello

**Affiliations:** 1 Department of Basic and Applied Sciences for Engineering, Sapienza University of Rome, Via del Castro Laurenziano 7, 00161 Rome, Italy; nichil.1741910@studenti.uniroma1.it; 2 National Center for Drug Research and Evaluation, Italian National Institute of Health, Viale Regina Elena, 299, 00161 Rome, Italy or sofia.migani@unicam.it (S.M.); marisa.colone@iss.it (M.C.); giuseppina.bozzuto@iss.it (G.B.); 3 School of Science and Technology, Chemistry Division, University of Camerino, Via Madonna delle Carceri (ChIP), 62032 Camerino, Macerata, Italy; 4 Institute for Complex Systems (ISC), National Research Council of Italy (CNR), c/o Department of Physics, Sapienza University of Rome, Piazzale Aldo Moro 5, 00185 Rome, Italy; leonardo.severini@roma1.infn.it (L.S.); simona.sennato@cnr.it (S.S.); 5 Institute for Biological Systems (ISB), National Research Council of Italy (CNR), Secondary Office of Rome-Reaction Mechanisms c/o Department of Chemistry, Sapienza University of Rome, Piazzale Aldo Moro 5, 00185 Rome, Italy; aurora.patrizi@sns.it (A.P.); cecilia.bombelli@cnr.it (C.B.); 6 Institute of Translational Pharmacology, National Research Council of Italy (CNR), Via Fosso del Cavaliere 100, 00133 Rome, Italy; gprav@unive.it

**Keywords:** polydatin, chitosan, nanoparticles, drug delivery, breast cancer, antiproliferative activity

## Abstract

Reactive oxygen species (ROS) are highly reactive molecules that, when produced in excess, contribute to oxidative stress, promoting cellular damage and the progression of various diseases, including cancer. Polydatin (PD) is known for its antioxidant, anti-inflammatory, and pro-apoptotic properties, proving effective in several in vitro studies as an antitumor agent. However, its clinical application is limited by low bioavailability, poor water solubility, and chemical instability. To overcome these limitations, nanocarrier systems based on biopolymers, such as chitosan (CS), represent promising strategies for drug delivery. In this study, we developed and optimized CS nanocapsules loaded with Polydatin using the ionotropic gelation method. The final formulation was characterized by UV-Vis spectrophotometry, scanning electron microscopy (SEM), and dynamic and dielectrophoretic light scattering (DLS, DELS). Encapsulation efficiency (EE) and the biological effects of the nanocapsules on cancer cells were also evaluated. To assess their antitumor potential, PD-CS nanoparticles were tested on the human breast cancer SKBR3 cells, analyzing their effects on cell viability. The results demonstrate that CS nanocapsules loaded with PD are able to reduce SKBR3 cell proliferation, highlighting their potential for developing new therapeutic tools for cancer treatment.

## 1. Introduction

Polydatin (PD, IUPAC name: (2*S*,3*R*,4*S*,5*S*,6*R*)-2-{3-hydroxy-5-[(1*E*)-2-(4-hydroxyphenylethenyl]phenoxy}-6-(hydroxymethyl)oxane-3,4,5-triol), also known as Piceid, is a glucoside of resveratrol in which a glucose molecule is linked to the hydroxyl group at C-3 [1]. This phytochemical is primarily found in *Fallopia japonica*, a plant used for centuries in traditional Chinese medicine, but it is also present in other plant sources such as red wine, nuts, vegetables, fruits, and cocoa. There are two isomeric forms of PD, cis and trans.

The limited bioavailability of resveratrol poses a significant challenge to its clinical utility. However, recent studies suggest that PD may offer superior bioavailability and more effective antioxidant activity compared to resveratrol itself [2,3,4,5].

Research indicates that PD exerts its effects through multiple mechanisms, including modulation of cell cycle regulators, induction of apoptosis, inhibition of metastasis, and alteration of oxidative stress pathways [6]. Reactive oxygen species (ROS), including superoxide anion (O_2_^−^), hydroxyl radical (•OH), and hydrogen peroxide (H_2_O_2_), are critical to cancer development and progression. Originating largely from mitochondria, ROS mediate cellular signaling and can promote tumorigenesis by enhancing proliferation, inducing genomic instability, suppressing apoptosis, and facilitating metastasis [7].

While basal levels of ROS are essential for tumor cell survival, excessive ROS levels become toxic, causing damage to carbohydrates, nucleic acids, lipids, and proteins [8]. Under normal physiological conditions, ROS are kept in check by the body’s antioxidant defense systems. However, when ROS levels surpass antioxidant capacity, oxidative stress ensues, contributing to the development of over 100 diseases, including cancer, neurodegenerative diseases, cardiovascular and inflammatory conditions [9].

This dual role of ROS in cancer has prompted increasing interest in phytochemicals with antioxidant capacity, particularly polyphenols, which are favored due to their low toxicity, good bioavailability, and multi-functional health benefits [1,2,10,11,12,13]. Resveratrol and its glucoside derivative, PD, have demonstrated chemopreventive and anticancer activity. In addition to its antioxidant properties, PD has demonstrated the ability to modulate apoptosis and exhibits significant anti-inflammatory effects, suggesting its therapeutic potential for chronic and degenerative diseases, including cancer [2].

In fact, in cervical cancer cells, PD has been shown to inhibit proliferation: inducing cell cycle arrest at the G0/G1 phase and suppression of metastasis by increasing E-cadherin and decreasing N-cadherin, Snail, and Slug expression, thereby reducing cell migration and invasion and downregulating c-Myc: The proto-oncogene c-Myc, associated with cell proliferation and metastasis, is significantly decreased following PD treatment, contributing to its anticancer effects [14]. In lung cancer, PD exhibits anticancer activity by inducing apoptosis through the activation of caspase pathways and causing DNA damage and cell cycle arrest [15]; PD is also used in chemotherapy to enhance the efficacy of cisplatin in non-small cell lung cancer (NSCLC) by inducing ROS generation and promoting apoptosis [16]. In colorectal and oral cancers, PD inhibits proliferation and promotes apoptosis by modulating the miR-382/PD-L1 axis [17]. In oral squamous cell carcinoma, PD induces mitochondrial-mediated apoptosis and inhibits cell migration and invasion by regulating EMT-related transcription factors [18].

Despite these promising pharmacological properties, PD’s clinical application is hampered by its low bioavailability, primarily due to poor water solubility, chemical instability in alkaline environments and extensive first-pass metabolism. To overcome these limitations and increase the selectivity of PD against cancer cells, nanoformulations have garnered significant interest in both academic and industrial pharmaceutical research. Nanoformulations, made of organic or inorganic nanostructures such as polymer nanoparticles, lipid nanoparticles, micelles and carbon nanotubes, serve as versatile, biocompatible, and biodegradable platforms for drug delivery. They can enhance the solubility, protect compounds from degradation, prolong drug release, and improve targeted delivery [19,20].

Among the various materials used in nanoformulations, biopolymers are notable for their biocompatibility and biodegradability. They can be natural or synthetic, with natural animal-derived biopolymers like albumin, gelatin, hyaluronic acid, and CS [21] being particularly attractive due to their abundance, low toxicity, and ease of use. CS, a polymer derived from the deacetylation of chitin, is highly valued for nano- and micro-delivery applications because of its hydrophilicity, low toxicity, and the ease with which it forms nanoparticles [22,23,24,25]. It can also be used to produce films, gels, beads, and fibers [26,27].

CS is available in a range of molecular weights, which affects its water solubility and nanoparticle properties. Low-molecular-weight CS is more hydrosoluble and thus more suitable for nanoparticle preparation. Additionally, its free amino groups allow for functionalization with other molecules, enhancing its utility in targeted therapies [23].

Polymeric nanocapsules, typically spherical in shape, can be synthesized through several methods. The most commonly used techniques for preparing CS nanocapsules include drying, polyelectrolyte complexation [28,29,30], nanoemulsification and ionotropic gelation [31,32], the latter being the most widely adopted due to its simplicity and does not involve the use of organic solvents, which is an important requirement for safety and for a possible scale-up [33,34,35].

In this work, PD-loaded CS nanoparticles were prepared using low-molecular-weight CS and the ionotropic gelation method [36]. After optimizing the process for maximum efficiency, the final formulation—a CS/PD 1:1 (*w*/*w*) ratio—was characterized by UV-Vis spectrophotometry, scanning electron microscopy (SEM), and dynamic light scattering (DLS). The encapsulation efficiency (EE) was also determined.

To evaluate the potential antiproliferative activity of the developed PD-loaded CS nanoparticles, the human breast cancer SKBR3 cells, which overexpress the HER2 receptor, were chosen as a model. Cell viability, mechanisms of cytotoxicity induction, and morphological changes were analyzed using fluorescence microscopy.

Some scientific publications have dealt with chitosan nanoparticle-based systems for polydatin delivery [37,38,39,40,41], but none of them have considered the application to breast cancer, to our knowledge.

## 2. Results

### 2.1. NPs Preparation

Empty (CS-NPs) and loaded (PD-CS-NPs) nanoparticles were prepared by an ionotropic gelation procedure, properly modified to improve NP polydispersity and the EE% of the PD. PD-loaded CS nanoparticles were prepared with a PD concentration of 0.5 mM, in a 1:1 ratio with CS (CS/PD, *w*/*w*). The pH of the dispersion during the preparation was controlled and set at pH 4 during the chitosan dissolution phase and below pH 5 after the addition of PD. Under these pH conditions, PD is stable and in neutral form, and is therefore encapsulated in the nanoparticles without participating in the gelation reaction, by physical entrapment in the CS-TPP network. Experimental details are reported in Section 4.

### 2.2. PD-CS-NPs Characterization

**Figure 1:**
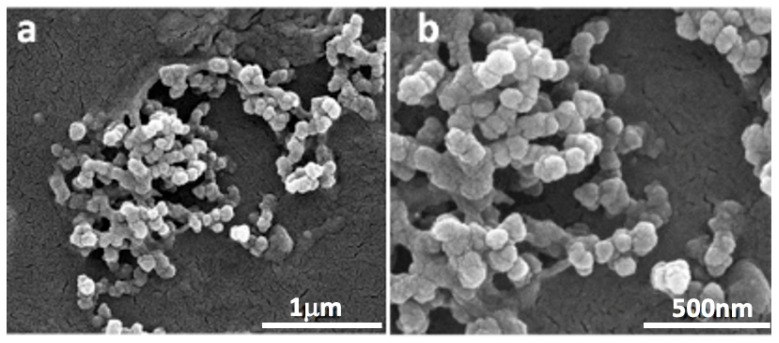
SEM micrographs of PD-loaded nanoparticles: magnification 80,000× (**a**); magnification 160,000× (**b**). The images were obtained using a FEI Quanta Inspect FEG SEM (FEI, Hillsboro, OR, USA) at 20 kV.

In order to characterize the PD–CS-NPs obtained from the optimization rounds, different analytical techniques have been employed. The morphology was observed by scanning electron microscopy. As is evident from the image (Figure 1), the spherical nanoparticles are in a highly aggregated state due to the air-dehydrated environment used to prepare SEM samples. This is also not surprising given their size in the range of 50–200 nm and the fact that the preparation process chosen was ionotropic gelation. Furthermore, similar morphology and aggregation patterns were observed in different studies concerning the preparation of CS nanoparticles.

**Figure 2:**
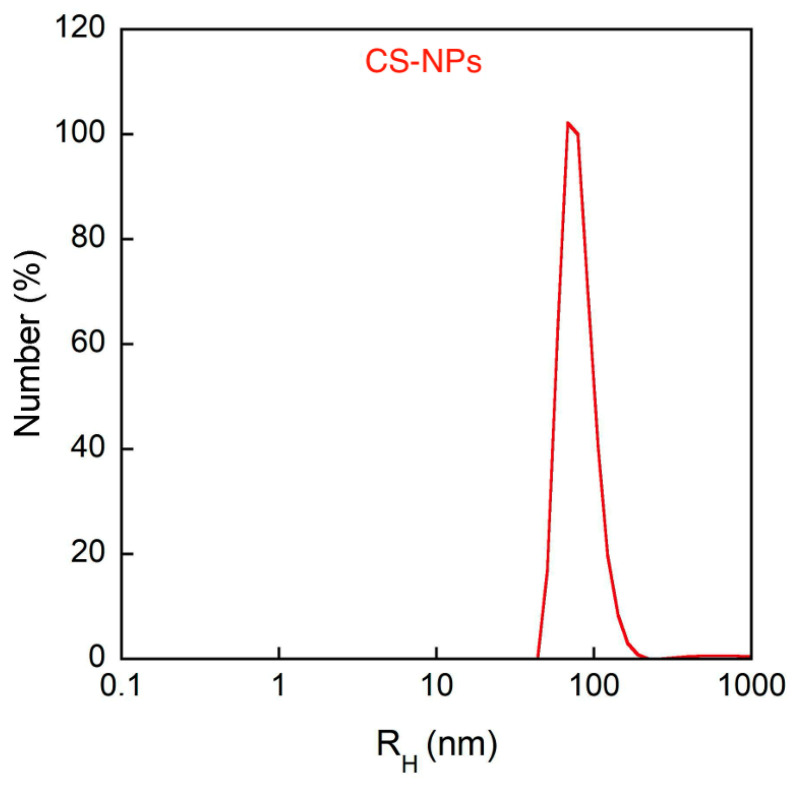
Number-weighted size distribution of dispersion of CS nanocapsules without PD (CS-NPs) obtained from DLS analysis.

**Figure 3:**
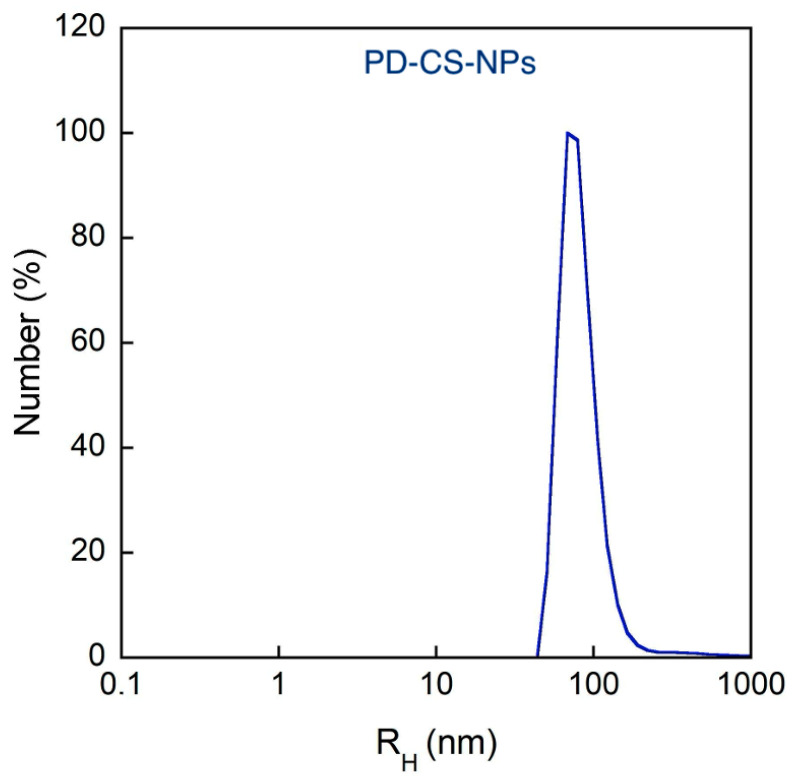
Number-weighted size distribution of dispersion of CS nanocapsules with PD (PD–CS-NPs).

Using the dynamic light scattering (DLS) technique, the average hydrodynamic size and the polydispersity index (PDI) of the samples were obtained by the cumulant method, as this represents the most straightforward method for size evaluation. However, PDI values exceed 0.4, and the analysis may lose significance, as the size distribution might exhibit multiple peaks (Table 1). To clarify this point, NNLS number-weighted analysis of DLS measurements was carried out, showing a mean hydrodynamic radius of about 100 nm for both CS-NPs and PD–CS-NPs (Figure 2 and Figure 3). The evaluation of these populations in NNLS number-weighted analysis is about 100%, demonstrating that they are almost the only ones present in the sample, according to SEM analysis. The ζ-potential of the CS-NPs nanocapsules is largely positive, as expected, and it is significantly reduced in the presence of PD, which may be indicative of a complexation between the drug and chitosan. In any case, we verified that both empty and loaded NPs remain stable in size and charge for at least 72 h.

The encapsulation efficiency and the loading capacity were evaluated by UV-Vis spectroscopy, determining the PD concentration in the dialysis medium. The nanoparticles obtained by mixing PD and CS in a 1:1 (*w*/*w*) showed percentages of 83% of EE using Equation (1) as reported in the Materials and Methods Section (Section 4) [42].

### 2.3. Evaluation of Antiproliferative Activity

**Figure 4:**
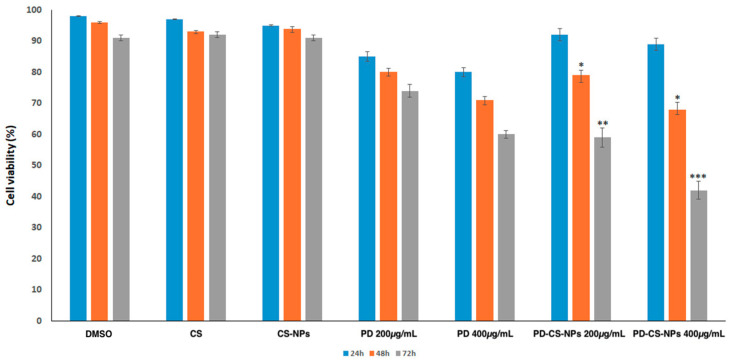
Cell viability analysis of SKBR3 cells treated with DMSO, CS, empty CS-NPs, PD, and PD–CS-NPs at concentrations of 200 and 400 μg/mL for 24, 48, and 72 h. Experiments were performed in triplicate, and results are presented as mean ± standard deviation. Statistical significance was evaluated using the t-test, with differences considered significant at *p* < 0.05 (*), *p* < 0.01 (**), and *p* < 0.001 (***).

The antiproliferative activity of PD–CS-NPs was tested on the human breast cancer SKBR3 cells. The MTT assay was performed on SKBR3 cells treated with PD alone and PD–CS-NPs at two different concentrations (200 and 400 μg/mL) for 24, 48, and 72 h (Figure 4). The results showed that after 24 h of treatment, neither concentration was able to induce a reduction in cell proliferative capacity. After 48 h, a decrease in cell viability of 20% was observed for treatments with PD–CS-NPs at a concentration of 200 μg/mL and 30% for treatments with PD–CS-NPs at 400 μg/mL. These results demonstrated that, although there was a slight decrease in viability, PD–CS-NPs were not yet cytotoxic to SKBR3 cells. In contrast, after 72 h, the nanocapsules were able to induce a reduction in the proliferative capacity of SKBR3 cells by 40% following treatment with PD–CS-NPs at 200 μg/mL and by 60% at 400 μg/mL. The empty chitosan nanocapsules, used as a control, were found to be highly biocompatible.

#### Evaluation of the Effects of PD–CS-NPs on the Actin Network and Morphology of SKBR3 Cells

**Figure 5:**
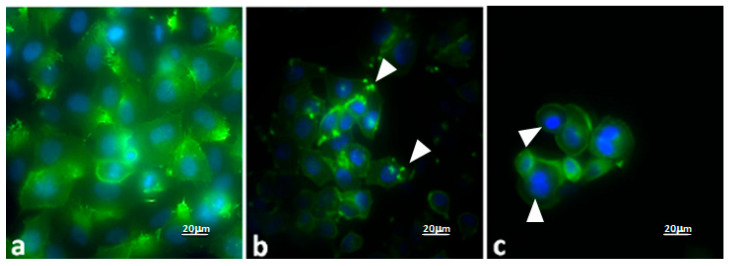
Micrographs obtained by fluorescence optical microscopy: (**a**) control SKBR3 cells; (**b**) SKBR3 cells treated for 72 h with PD–CS-NPs at a concentration of 200 μg/mL; (**c**) SKBR3 cells treated for 72 h with PD–CS-NPs at a concentration of 400 μg/mL. Actin is stained in green, while the nucleus appears blue.

To understand the mechanism by which PD–CS-NPs induce a noticeable effect on the cell viability of the SKBR3 line, the cells were treated for 72 h at the two tested concentrations and observed under a fluorescence microscope. The control SKBR3 cells (Figure 5a) exhibit their typical polygonal morphology without any evident alterations in the cytoskeleton. As shown in Figure 5a, most of the control cells have a highly organized actin cytoskeleton profile composed of numerous intact actin filaments. Moreover, in SKBR3 cancer cells, chromatin appears regular within the nucleus. Numerous SKBR3 cells are adherent to the coverslip. SKBR3 cells treated with PD–CS-NPs at a concentration of 200 μg/mL for 72 h (Figure 5b) show minimal chromatin organization and morphological cell alterations. Interestingly, in SKBR3 cells, the PD–CS-NPs treatment induced significant actin depolymerization. Additionally, fewer cells are observed adhering to the substrate, but they display numerous focal adhesion points (arrowheads). Instead, SKBR3 cells treated with PD–CS-NPs at a concentration of 400 μg/mL for 72 h are almost completely detached from the substrate with evident morphological alterations due to antiproliferative activity induced by nanocapsule treatment. Cells also showed the condensation of chromatin leading to the induction of apoptosis (Figure 5c, arrowheads). These results are consistent with the MTT results (see above).

## 3. Discussion

Polydatin, also known as piceid, is a natural glycosylated form of resveratrol found primarily in plants such as *Fallopia japonica*. In recent years, PD has attracted considerable interest in biomedical research due to its promising pharmacological properties, particularly its antioxidant, anti-inflammatory, and antitumor activities. One of the main challenges in utilizing PD for therapeutic purposes is its low bioavailability, which limits its clinical efficacy. Therefore, the development of advanced drug delivery systems has become essential to enhance its absorption, stability, and targeted delivery. Nanoparticle-based delivery systems, liposomes, and polymeric micelles have been explored as effective carriers for PD. These systems not only improve its bioavailability but also allow for controlled release and targeted delivery to tumor tissues, minimizing systemic side effects. Regarding its antitumor effects, PD has demonstrated potential against various types of cancers, including breast, lung, liver, and colorectal cancers [43,44]. Its mechanisms of action involve the induction of apoptosis (programmed cell death), inhibition of cell proliferation, reduction in angiogenesis (formation of new blood vessels that supply tumors), and modulation of key signaling pathways such as PI3K/Akt, MAPK, and NF-κB. Moreover, PD shows the ability to reduce oxidative stress in cancer cells and sensitize them to conventional chemotherapy, potentially overcoming drug resistance.

In combination with advanced drug delivery systems, polydatin’s therapeutic efficacy in oncology can be significantly improved. By enhancing its stability and targeting ability, these delivery systems increase polydatin’s accumulation in tumor tissues, thereby maximizing its antitumor activity while reducing toxicity to healthy tissues. Encapsulation improves the delivery of PD to breast cancer cells by increasing its solubility and stability in biological fluids. Nanocarriers can facilitate endocytosis-mediated uptake by cancer cells, allowing for a higher intracellular concentration of PD compared to its free form. Additionally, surface modifications on nanocarriers can be used to achieve active targeting of breast cancer cells by recognizing specific receptors (e.g., folate receptors, HER2) [45,46].

Studies have shown that encapsulated PD exhibits stronger cytotoxic effects against breast cancer cells compared to free PD This enhanced activity is due to a combination of higher drug accumulation, better cellular uptake, and prolonged exposure of cancer cells to the active compound. Encapsulated PD more effectively induces apoptosis, arrests the cell cycle, and reduces cancer cell proliferation [47].

Another advantage of encapsulated formulations is the reduced systemic toxicity. By directing PD specifically to breast cancer cells, encapsulated systems minimize exposure to healthy tissues, decreasing potential side effects commonly associated with anticancer treatments. Encapsulation of PD represents a promising strategy to overcome the pharmacological limitations of free PD. By improving solubility, bioavailability, targeting ability, and therapeutic efficiency, encapsulated PD demonstrates stronger antitumoral effects on breast cancer cells while minimizing adverse effects. This approach holds significant potential for the development of more effective, natural compound-based therapies in breast cancer treatment.

Despite the promising properties of the PD, as written above, its activity is often hampered because of its instability, so that its incorporation/protection into nanocarriers may represent the most promising strategy. In light of this, in the present study, biocompatible nanovectors, such as chitosan nanocapsules, have been formulated for the effective incorporation of the PD bioactive molecules. These systems are also capable of avoiding the rapid degradation of the PD, facilitating its uptake into human cells, especially in cancer cells.

The identified preparation protocol had to satisfy the following requirements: high encapsulation efficiency, which guarantees the optimal PD/CS ratio; an average size of the nanocapsules less than or equal to 100 nm, to ensure effective interaction with cells and the possible overcoming of biological barriers; a surface charge equal to or greater than |30 mV|, to guarantee good stability of the system.

Using UV-Vis spectroscopy, an entrapment efficiency of 83% was indirectly calculated by determining, in the dialysis medium, the amount of polydatin not encapsulated in the nanoparticles. The calculation was based on an initial PD concentration of 0.5 mM in a 5 mL solution used for the synthesis, and a PD concentration of 13.8 μM measured in a 31 mL solution obtained from the dialysis water, thus giving a final PD concentration of 0.42 mM. NNLS intensity-weighted analysis of DLS measurements showed a mean hydrodynamic radius of about 100 nm for PD–CS-NPs, thus validating the predefined size target.

We have demonstrated that PD–CS-NPs can more effectively reduce cell proliferation and induce cell death in human breast cancer SKBR3 cells. Indeed, PD–CS-NPs can induce a significant antiproliferative effect as evidenced by the cell viability assay (Figure 5) when compared to free PD and unloaded CS nanoparticles. Moreover, PD–CS-NPs at a concentration of 400 μg/mL revealed a higher cytotoxic effect on the SKBR3 cell survival than those loaded with 200 μg/mL PD, indicating that these results are both dose- and time-dependent. PD’s cytotoxic effect was ensured by the loading of the PD-CS-NPs, which likely promotes more efficient interaction with the cancer cell membrane and facilitates internalization into the cytoplasm, where PD induces cellular damage as demonstrated by immunofluorescence observations (Figure 5). These findings suggest that this drug delivery system enhances the desired biological effects of PD, as it demonstrates antiproliferative activity against human breast cancer SKBR3 cells. These results are encouraging but they should be interpreted as a proof-of-concept demonstration limited to in vitro conditions. Further studies, particularly in vivo, will be necessary to confirm the therapeutic potential and safety profile of this system.

## 4. Materials and Methods

### 4.1. Chemicals

CS oligomer (CS) at ultra-low molecular weight (MW: 1526.464) (Fluorochem Ltd., Hadfield, UK) was used in this study. PD (PD) (Piceid, CAS RN: 27208-80-6) was purchased from Tokyo Chemical Industry Co., Ltd. (Tokyo, Japan). A dialysis membrane (Spectra/Por Biotech CE Trial Kit; MWCO: 300 kDa; Carlo Erba Reagents srl, Milan, Italy) was used for the experiments. All other chemicals unless specified, were purchased from Merck Life Science srl, Milan, Italy.

### 4.2. Nanoparticles Preparation

Ionotropic gelation is a technique used for the formation of micro- or nanoparticles, based on electrostatic interactions between oppositely charged species, typically a cationic polymer and an anionic species. The latter acts as a cross-linking agent, serving as a bridge between polymer chains and promoting the formation of the gel’s three-dimensional network.

In our case, the cationic polymer employed is chitosan (CS). Under acidic conditions, the free amino groups present on the glucosamine residues become protonated (−NH_3_^+^), imparting a positive charge to chitosan and making it a polycationic polymer. Due to this property, chitosan can electrostatically interact with polyanionic molecules, leading to the formation of nanocapsules.

The cross-linking of chitosan occurs in a solution containing the drug to be encapsulated: the liquid phase becomes entrapped within the polymeric shell and is subsequently released upon polymer degradation. Among the most commonly used anionic species serving as cross-linkers are polyphosphate salts, which completely dissociate in aqueous solution, releasing the polyphosphate anion responsible for forming ionic bridges between the chitosan chains.

Among these, one of the most widely used is sodium tripolyphosphate (TPP, Na_5_P_3_O_10_), which was also employed in the present study for the synthesis of chitosan nanocapsules using the ionotropic gelation method.

The total volume of nanoparticles to be prepared was set to 5 mL. The required amounts of the various components—chitosan, salt (NaCl), sodium tripolyphosphate (TPP), and polydatin (PD)—were calculated, weighed, and stored in appropriate vials.

- Chitosan: To obtain a final concentration of 0.5 mg/mL, 2.5 mg of the polymer was weighed.
- Salt (NaCl): The same final concentration of 0.5 mg/mL was maintained, so 2.5 mg of salt was weighed.
- TPP: A weight ratio of 6:1 between chitosan and TPP was selected. Applying the ratio 6:1 = 2.5:x, the calculated value was x = 0.0005 g, corresponding to 0.5 mg of TPP. The TPP was then dissolved in 700 μL of deionized water to obtain a solution with a concentration of 0.7 mg/mL.
- Polydatin (PD): A final concentration of 0.5 mM was chosen. This concentration was determined based on a solubility study of polydatin in water, which showed that, at the same pH, higher concentrations were not possible. To prepare the solution, a volume of 2.5 mL of PD solution in deionized water at 1 mM concentration and pH 4.8 was prepared. Considering that the final total volume would be 5 mL, the concentration would decrease by half, reaching the desired final concentration. The solution was prepared by adding 2.465 mL of deionized water to 0.001 g of polydatin and sonicating the mixture in an ultrasonic bath for 30 min at 40 °C. Subsequently, 0.035 mL of a 1 mM acetic acid solution in deionized water was added to adjust the pH to 4.8 and reach the final volume of 2.5 mL. The preparation was completed with a second sonication step for 10 min at 25 °C.

Next, the previously weighed chitosan was placed into a 250 mL round-bottom flask positioned on a magnetic stirrer. A volume of deionized water was added, calculated according to the formula:

V = V_tot_ − V_PD_ − V_TPP_

where V_tot_ represents the total nanoparticle volume (5 mL), V_PD_ the volume of the polydatin solution, and V_TPP_ the volume of the TPP solution.

The system was stirred at 365 rpm. Then, 10 μL of acetic acid was added (down to pH 4), and stirring was maintained for 10 min to ensure complete solubilization of the chitosan. At this point, the previously weighed NaCl was dissolved in a small portion of the solution and then added to the main system. Stirring was continued for 4 h to ensure complete homogeneity and equilibrium of the mixture.

After 4 h, the polydatin solution was added, and the stirring speed was increased to 500 rpm, maintaining agitation for 1 h. To further improve PD solubilization, the flask was placed in an ultrasonic bath for 10 min at room temperature. After this treatment, the flask was repositioned on the magnetic stirrer, and the stirring speed was returned to 365 rpm.

**Figure 6:**
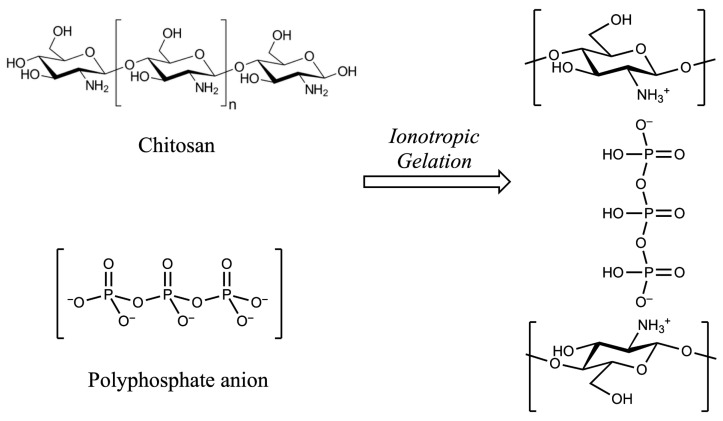
Schematic representation of the ionotropic gelation of chitosan and polyphosphate anion.

Under these conditions, the TPP solution was added gradually, 100 μL at a time, with one-minute intervals between additions. Once all the TPP solution had been added, stirring was maintained for an additional 20 min to allow complete formation of the chitosan nanocapsules through the ionotropic gelation process (Figure 6).

After synthesis, a dialysis process was carried out to remove unencapsulated polydatin. The dialysis medium volume was set to fifty times the total sample volume. The dialysis solution was prepared with the same NaCl concentration present in the nanoparticles to maintain isotonicity.

Before use, the dialysis membrane was activated by cutting a piece of the desired length and immersing it for 30 min in a beaker containing deionized water. Subsequently, the membrane was rinsed with a solution of deionized water and NaCl at the same concentration as in the sample to ensure ionic compatibility.

**Figure 7:**
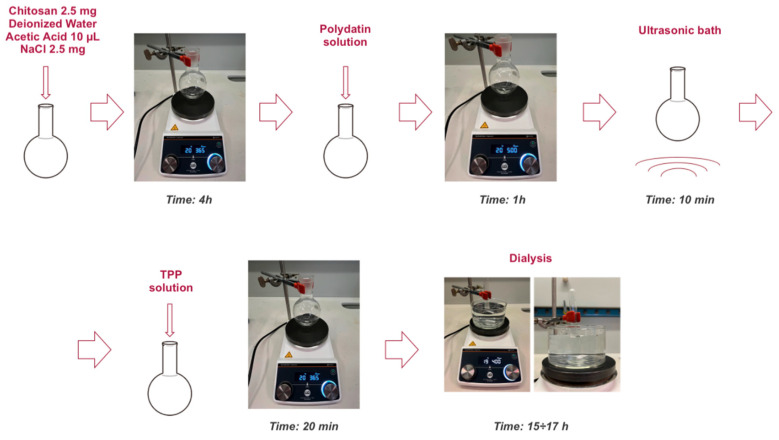
Schematic representation of nanocapsule preparation.

The nanoparticle sample was then placed into a vial, sealed with the activated membrane, and secured with parafilm. Dialysis was carried out for 15–17 h, positioning the vial upside down so that it was in contact with the surface of the dialysis solution (Figure 7).

### 4.3. Nanoparticles Characterization

#### 4.3.1. Scanning Electron Microscopy

PD-loaded CS nanocapsules were seeded onto 12 mm circular glass coverslips pre-coated with poly-L-lysine to promote adhesion. After 30 to 60 min, the coverslips were air-dried and subsequently subjected to sputter coating. During this process, the samples were exposed to a stream of ionized inert gas particles (Ar) that bombarded a metallic target (Au), causing superficial erosion of the target material. The eroded gold particles were then deposited onto the sample surface, forming a uniform conductive coating.

The sputtering procedure was carried out using a Balzers SCD 040 coater (Bal-Tec, Balzers, Liechtenstein), which consists of a vacuum chamber equipped with a sample holder and a gas inlet system that allows controlled introduction of the inert gas. Once the desired vacuum was achieved, the discharge was initiated to generate a cloud of gold particles that coated the samples.

After coating, the coverslips were mounted on aluminum stubs using silver adhesive to ensure proper chemical–physical stability and electrical conductivity, both essential for acquiring interpretable images. The prepared samples were then examined using a field emission scanning electron microscope (FEI Quanta Inspect FEG, FEI, Hillsboro, OR, USA) operated at an accelerating voltage of 30 kV.

#### 4.3.2. Dynamic and Dielectrophoretic Light Scattering

Dynamic and Dielectrophoretic light scattering (DLS, DELS) measurements for determination of size and ζ-potential, respectively, were performed by the Nano Zetasizer ZS instrument (Malvern Instrument, Malvern, UK), equipped with a polarized monochromatic beam emitted by a solid-state laser (100 mW; λ = 642 nm) and a thermostated cell holder. For DLS measurement, scattered light is collected at an angle θ = 173°, which, according to the relation q = 4πn/λ sin (θ/2), corresponds to a scattering vector q = 0.018 nm^−1^. The autocorrelation functions have been first analyzed by the cumulant method, to obtain the average hydrodynamic size and the polydispersity index (PDI) of the samples, since this is the most direct way for the determination of the size [48]. However, when PDI values are larger than 0.2–0.3, this analysis may not be significant because the size distribution could show more than one maximum. To ascertain this point, a volume-weighted NNLS algorithm has been used, in the framework of the Mie scattering theory dispersion to determine the whole size distribution [49]. For this analysis, the refractive index of nanoparticles has been approximated with that of polystyrene.

#### 4.3.3. Evaluation of Encapsulation Efficiency

The dialysis waters were evaporated to dryness using a rotary evaporator set at a temperature of 50 °C and a rotation speed of 100 rpm. Once dried, ethanol was added to the flask. The supernatant was collected and centrifuged at 10,000 rpm for 10 min at 25 °C. After centrifugation, the supernatant was evaporated to dryness again, diluted with 31 mL of deionized water, and analyzed using a spectrophotometer at a wavelength of 320 nm [50]. The encapsulation efficiency (EE) of PD was calculated as follows:

(1) $$\mathrm{EE}=\frac{n^{*}-n}{n^{*}}\times 100,$$

where “n*” represents the moles of PD used for the synthesis, and “*n*” represents the moles of PD present in the dialysis waters, meaning not encapsulated.

### 4.4. Cell Cultures

The cell line used for cytotoxicity tests is the human breast cancer SKBR3 cells, provided by the American Type Culture Collection (ATCC: the Global Bioresource Center, Manassas, VA, USA) (Figure 4). This cell line, derived from a metastatic human mammary adenocarcinoma, was first isolated in 1970 at the Memorial Sloan–Kettering Cancer Center in New York from a pleural effusion obtained from a 43-year-old Caucasian woman with breast adenocarcinoma. SKBR3 cells are characterized by the overexpression of the HER2 receptor (human epidermal growth factor receptor 2), a tyrosine kinase crucial in the pathogenesis and progression of certain types of breast cancer.

The cells were cultured in RPMI 1640 (Roswell Park Memorial Institute) media (EuroClone, Pero, Milan, Italy), supplemented with 10% fetal bovine serum (FBS South America, Corning, NY, USA), 1% penicillin-streptomycin, 1% L-glutamine and 1% non-essential amino acids (EuroClone). The cells were maintained at 37 °C in a humidified atmosphere with 5% CO_2_ and sub-cultured at confluence using trypsin/EDTA.

### 4.5. Assessment on PD-CS-NPs Activity

#### 4.5.1. MTT Assay

SKBR3 cells were seeded in 96-well microplates (EuroClone, Pero, Milan, Italy) at a density of 10 × 10^4^ cells per well. After 24 h of adhesion at 37 °C with 5% CO_2_, the cells were treated with nanocapsules with two different concentrations (200 and 400 μg/mL) and were incubated for 24, 48, and 72 h. At the end of each period, the cells were incubated with a 0.5 mg/mL MTT solution (Sigma-Aldrich) for 3 h at 37 °C. MTT salt was dissolved in PBS at a concentration of 0.5 mg/mL, and 100 μL of the MTT-containing solution was added. The plates were then incubated again at 37 °C with 5% CO_2_ for 3 h. Subsequently, the MTT-containing medium was removed, and formazan was solubilized with 100 μL of DMSO.

#### 4.5.2. Cellular Vitality

Absorbance measurements were performed using the Varioskan™ LUX multimode plate spectrophotometer (Thermo Fisher Scientific, Waltham, MA, USA) at a wavelength of 570 nm. The results were expressed as the ratio of the absorbance of the treated sample to that of the untreated control:

(2) $$\mathrm{Cellular}\;\mathrm{vitality}\;(\%)=\frac{O.D.\;of\;treated\;cells}{O.D.\;of\;untreated\;cells}\times 100$$

where O.D. represents the optical density of the sample read at 570 nm absorbance.

#### 4.5.3. Statistical Analysis

The MTT assay was independently repeated three times. The results were then presented as mean ± standard deviation. Statistical analysis was performed using the *t*-test, and differences were considered significant for *p* < 0.05 (*), *p* < 0.01 (**), and *p* < 0.001 (***).

#### 4.5.4. Fluorescence Microscopy

To understand the mechanism by which PD–CS-NPs induce a noticeable effect on the cell viability of the SKBR3 cell line, the cells were treated at the two tested concentrations and observed, after appropriate processing, under a fluorescence microscope to study the effects on the cytoskeleton and nucleus.

SKBR3 cells were seeded at a concentration of 3 × 10^4^ on 12 mm diameter coverslips for 24 h. The cells were then treated for 72 h with PD–CS-NPs at concentrations of 200 μg/mL and 400 μg/mL. The treated cells were fixed with 4% paraformaldehyde in PBS for 30 min and subsequently stained for actin detection using FITC-phalloidin (Sigma) 1:100 in PBS for 30 min at room temperature. For nuclear staining, the cells were labeled with the specific nuclear dye Hoechst 33258 (Sigma-Aldrich, St. Louis, MO, USA; 861405) at 37 °C for 15 min. After washing with PBS, the coverslips were mounted with PBS-glycerol, and images were acquired using a fluorescence microscope (Nikon Ti2 Eclipse, Nikon, Tokyo, Japan).

## Figures and Tables

**Table 1:** Physico-chemical properties of CS-NPs and PD-CS-NPs samples. Rh and PDI are the hydrodynamic diameter and the polydispersity index, respectively, calculated by the cumulants method; EE% is the entrapment efficiency determined by the ratio of PD concentration after and before dialysis, assessed by UV measurements [Sample name; Average radius; Polydispersity index (PDI), ζ-potential, Entrapment Efficiency].

Sample	R_H_ [nm] (Cumulants)	PDI (Cumulants)	ζ-Pot [mV]	EE%
CS-NPs	207 ± 8	0.65 ± 0.07	48 ± 1	-
PD-CS-NPs	68 ± 5	0.44 ± 0.08	5 ± 2	83

## Data Availability

All the data produced in this study are reported in this article. The primary data files are available from the corresponding authors upon reasonable request.
